# Crystal structures of three *N*-acyl­hydrazone isomers

**DOI:** 10.1107/S2056989021006885

**Published:** 2021-07-09

**Authors:** H. Purandara, Sabine Foro, B. Thimme Gowda

**Affiliations:** a Department of Chemistry, Mangalore University, Mangalagangotri-574 199, Mangalore, India; b Department of Chemistry, Sri Dharmasthala Manjunatheshwara College (Autonomous), Ujire 574 240, India; c Institute of Materials Science, Darmstadt University of Technology, Alarich-Weiss-Str. 2, D-64287, Darmstadt, Germany; d Karnataka State Higher Education Council, Y. Ramachandra Road, Gandhingar, Bengaluru-560009, India

**Keywords:** crystal structure, *N*-acyl­hydrazone, dihedral angle, inversion dimers, C—H⋯O inter­action

## Abstract

The crystal structures of three isomers of (*E*)-4-chloro-*N*-{2-[2-(chloro­benzyl­idene)hydrazin­yl]-2-oxoeth­yl}­benzene­sulfonamide, namely, (*E*)-4-chloro-*N*-{2-[2-(2-chloro­benzyl­idene)hydrazin­yl]-2-oxoeth­yl}­benzene­sulfonamide (I), (*E*)-4-chloro-*N*-{2-[2-(3-chloro­benzyl­idene)hydrazin­yl]-2-oxoeth­yl}­benzene­sul­fon­amide (II) and (*E*)-4-chloro-*N*-{2-[2-(4-chloro­benzyl­idene)hydrazin­yl]-2-oxo­eth­yl}­benzene­sulfonamide (III), with the general formula C_15_H_13_Cl_2_N_3_O_3_S are described, with the chloro group in *ortho*, *meta* and *para* positions in the benzyl­idene benzene ring. All the three isomeric compounds crystallize in the centrosymmetric triclinic *P*  space group with one mol­ecule each in the asymmetric unit and two mol­ecules in the unit cell. The dihedral angles between the two phenyl rings are 11.09 (14), 53.79 (18) and 72.37 (11)° in (I), (II) and (III), respectively. The central part of the mol­ecule (–C—N—N=C–) is almost linear with C—N—N—C torsion angles of 179.1 (2), −169.5 (3) and 178.5 (2)° for (I), (II) and (III), respectively. In all the three crystals, the mol­ecules form inversion dimers with *R*_2_^2^(8) ring motifs, which are further augmented by C—H⋯O inter­actions.

## Abstract 2

The crystal structures of three isomers of (*E*)-4-chloro-*N*-{2-[2-(chloro­benzyl­idene)hydrazin­yl]-2-oxoeth­yl}­benzene­sulfonamide, namely, (*E*)-4-chloro-*N*-{2-[2-(2-chloro­benzyl­idene)hydrazin­yl]-2-oxoeth­yl}­benzene­sulfonamide (I), (*E*)-4-chloro-*N*-{2-[2-(3-chloro­benzyl­idene)hydrazin­yl]-2-oxoeth­yl}­benzene­sulfonamide (II) and (*E*)-4-chloro-*N*-{2-[2-(4-chloro­benzyl­idene)hydrazin­yl]-2-oxoeth­yl}­benzene­sulfonamide (III), with the general formula C_15_H_13_Cl_2_N_3_O_3_S are described, with the chloro group in *ortho*, *meta* and *para* positions in the benzyl­idene benzene ring. All the three isomeric compounds crystallize in the centrosymmetric triclinic *P* 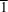 space group with one mol­ecule each in the asymmetric unit and two mol­ecules in the unit cell. In all the three crystals, the mol­ecules form inversion dimers with 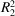 (8) ring motifs, which are further augmented by C—H⋯O inter­actions.

## Chemical context   

The properties of mol­ecules in solution and the solid state are strongly influenced by weak non-covalent inter­actions. Weak mol­ecular inter­actions are investigated routinely in the areas of mol­ecular recognition (Brouwer *et al.*, 1999 ▸), self-assembly (Seth *et al.*, 2011 ▸), supra­molecular chemistry and general host–guest inter­actions (Kim *et al.*, 2000 ▸; Sharma *et al.*, 2009 ▸). Analysis of inter­molecular inter­actions and estimation of their energies provide greater insights into mol­ecular conformations (Cao *et al.*, 2020 ▸; Jablonski, 2020 ▸). The nature and site of substituents influence the extent of polarization of electron distribution in covalent compounds. In our previous work (Purandara *et al.*, 2017*a* ▸,*b* ▸), the presence of the electron-withdrawing nitro group on the benzene ring was found to decrease the electronic density, rendering aromatic C—H protons acidic, whereas a methyl substituent did not activate aromatic protons for participation in inter­molecular C—H⋯O inter­actions. In a continuation of these efforts to study the effect of substituents on weak mol­ecular inter­actions, we report herein the synthesis, characterization and crystal structures of three isomeric mol­ecules. 
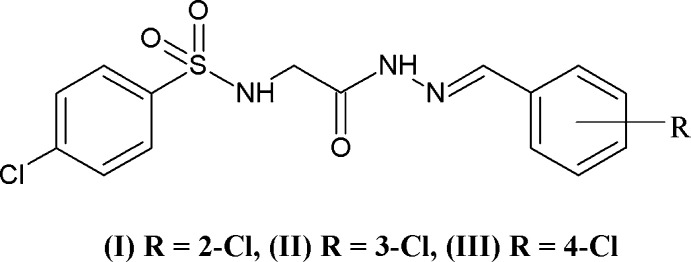


## Structural commentary   

**Figure 1:**
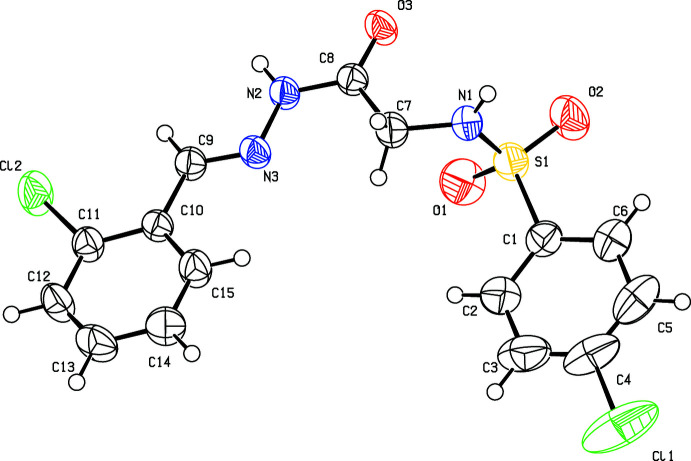
Mol­ecular structure of (I), showing the atom labelling and displacement ellipsoids drawn at the 50% probability level.

All three isomers (I)–(III) (Figs. 1 ▸–3 ▸ ▸) crystallize in the centrosymmetric triclinic system with space group *P* 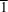 and with one mol­ecule in the asymmetric unit and two mol­ecules in the unit cell. The conformation of both the sulfonamide and hydrazine N—H bonds are *syn* with respect to the C=O bonds in all the three compounds. Similarly, the imine C—H bond in the amide part is also *syn* with respect to the amide N—H bond. All four such bonds in the central part are *syn* to each other. The C8—O3 and C9—N3 bond lengths of 1.224 (3)–1.236 (3) Å and 1.271 (3)–1.275 (4) Å, respectively, are in the ranges of normal C=O and C=N bond lengths, indicating double-bond character and thus confirming the keto tautomeric form and are comparable with those in related *N*-acyl­hydrazone structures (Purandara *et al.*, 2017 ▸). The delocalization of π-electron density over the C9/N3/N2/C8/O3 fragment is indicated by the shortening of the C8—N2 [1.337 (3)–1.342 (4) Å] distances compared to the normal C—N single bond length of 1.40 Å (Allen *et al.*, 1987 ▸). The sulfonamide bonds are synclinal, anti­clinal and anti­periplanar with the S1—N1—C7—C8 torsion angle being 83.6 (3), −107.2 (3) and 171.09 (18)°, in compounds (I), (II) and (III), respectively. The major twist in the mol­ecule occurs about the S1—N1 bond [C1—S1—N1—C7 = 97.6 (2) (I), 65.6 (2) (II) and −80.1 (2)° (III)], giving the mol­ecule an approximate overall L-shape. All the mol­ecules adopt an *E* configuration around the C9=N3 bond as indicated by the N2—N3—C9—C10 torsion angles of 179.6 (2), 179.1 (3) and 180.0 (2)° for (I), (II) and (III), respectively. The central fragment of the mol­ecule, (C9/N3/N2/C8/O3) is nearly coplanar with the phenyl ring (C10–C15), as indicated by the dihedral angles between their best planes of 4.2 (2) in (I), 11.9 (3) in (II) and 7.0 (3)° in (III). The dihedral angles between the two phenyl rings, C1–C6 and C10–·C15 are 11.1 (1), 53.8 (1) and 72.4 (1)° for (I), (II) and (III), respectively, indicating non-planarity of the three mol­ecules. An intra­molecular hydrogen bond, N1—H1*N*⋯O3, is observed in (II) and (III), generating an *S*(5) ring motif.

## Supra­molecular features   

**Figure 4:**
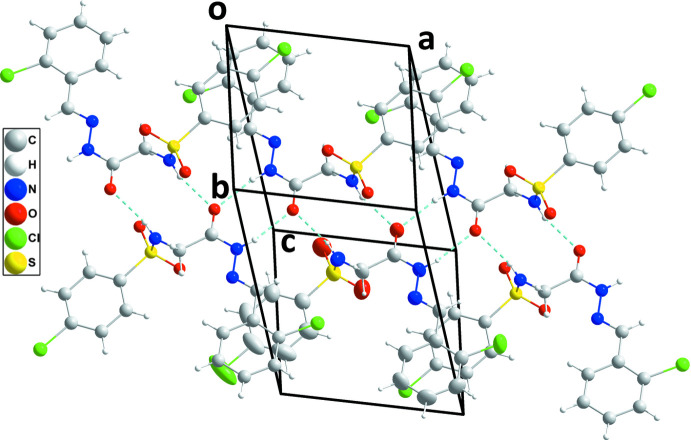
A view of a portion of one chain of inversion dimers in (I) connected by N—H⋯O hydrogen bonds (dashed lines) and extending along the *a*-axis direction.

**Figure 5:**
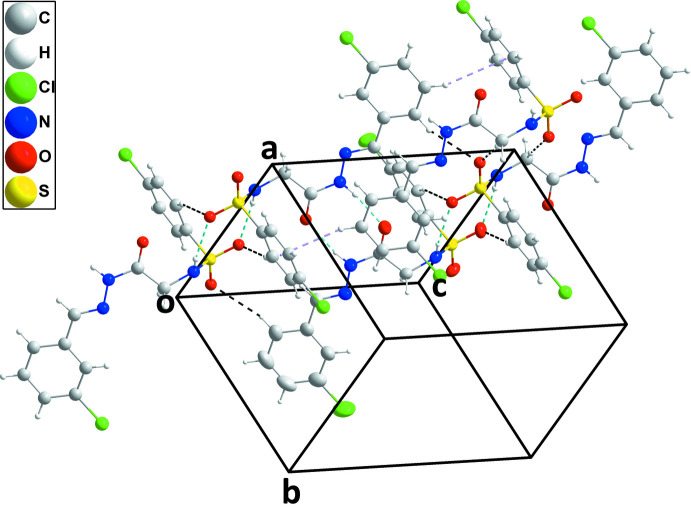
A partial packing diagram for (II) with N—H⋯O and C—H⋯O hydrogen bonds depicted, respectively, by light-blue and black dashed lines. The C—H⋯π(ring) inter­actions are depicted by violet dashed lines.

**Figure 6:**
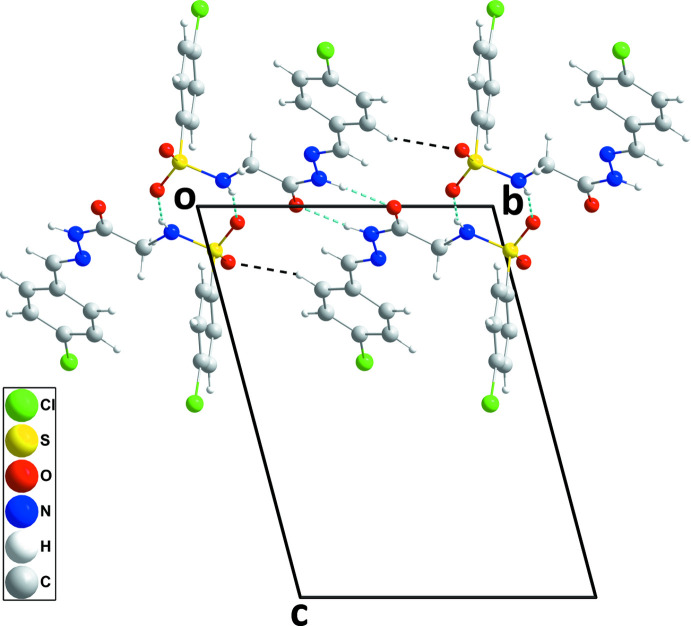
A portion of one chain in (III) viewed along the *a*-axis direction with hydrogen bonds depicted as in Fig. 5 ▸.

In the crystal of (I), the carbonyl oxygen (O3) shows bifurcated hydrogen bonding. In one part, the mol­ecules are linked by a pair of N2—H2*N*⋯O3 hydrogen bonds involving the amide NH atom, forming inversion dimers with an 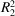 (8) ring motif. In the other part, the mol­ecules are linked by a pair of N1—H1*N*⋯O3 hydrogen bonds with the sulfonamide NH atom of another mol­ecule, forming rings with an 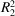 (10) graph-set motif, leading to a layered structure with the mean planes of the layers inclined to the *ab* plane by 16.1 (5)° (Table 1 ▸, Fig. 4 ▸). In the crystal of (II), the mol­ecules are linked by two pairs of N—H⋯O hydrogen bonds (N1—H1*N*⋯O2 and N2—H2*N*⋯O3), involving both the sulfonyl and carbonyl O atoms with both sulfonamide and amide N—H bonds (N1—H1*N* and N2—H2*N*), forming inversion dimers with 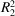 (8) ring motifs. These inter­actions are further strengthened by C—H⋯O hydrogen bonds. Thus, three-center N1—H1*N*/C6—H6⋯O1 hydrogen bonds result in mol­ecular chains containing the 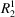 (7) ring motif (Fig. 5 ▸). These rings are extended along the principal diagonal of the *ac* plane *via* C7—H7⋯O2 hydrogen bonds, forming 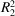 (10) ring motifs, and by C15—H15⋯O2 inter­actions. In addition, the crystal structure is reinforced by C—H⋯π(ring) inter­actions (Fig. 5 ▸), details of which are summarized in Table 2 ▸. In the crystal of (III), the mol­ecules are also linked by two pairs of N—H⋯O hydrogen bonds (N1—H1*N*⋯O2 and N2—H2*N*⋯O3), forming inversion dimers with 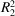 (8) ring motifs. These dimers are connected by inter­molecular C15—H15⋯O1 inter­actions, forming ribbons two mol­ecules wide and extending along the principal diagonal of the *ab* plane (Table 3 ▸, Fig. 6 ▸). The presence of the chlorine atom on the phenyl ring (C10–C15) of (I)–(III) makes the aromatic protons acidic, resulting in the formation of C—H⋯O hydrogen bonds with the sulfonyl O atom.

## Database survey   

Comparison of structures (I)–(III) with those of related *N*-acyl­hydrazone derivatives (Purandara *et al.*, 2017 ▸, 2018 ▸) shows that the site of substitution of an electron-withdrawing group on the aromatic ring plays a major role in stabilizing the crystal packing by linking the mol­ecules through various weak inter­actions.

## Synthesis and crystallization   

**General procedure for the synthesis of** ***N*** **-(4-chloro­benzene­sulfon­yl) glycine hydrazone derivatives (I)–(III)**

A mixture of *N*-(4-chloro­benzene­sulfon­yl) glycinyl hydrazide (0.01 mol) and the appropriate chloro­benzaldehyde (0.01 mol) in anhydrous methanol (30 mL) and two drops of glacial acetic acid was refluxed for 8 h. After cooling, the precipitate was collected by vacuum filtration, washed with cold methanol and dried. It was recrystallized to constant melting point from methanol. The purity of the compound was checked by TLC and characterized by its IR and NMR spectra. Single crystals suitable for the X-ray diffraction study were grown from DMF solution by slow evaporation of the solvent.

**Compound (I):** Prism-like yellow single crystals; m.p. 506–507 K; IR (KBr, γ, cm^−1^): 3190.3 (N—H), 1672.3 (C=O), 1608.6 (C=N), 1334.7 (S=O, asym) and 1159.2 cm^−1^ (S=O, sym); ^1^H NMR (400 MHz, DMSO-*d*_6_, *δ* ppm): 3.64, 4.14 (*d*, 2H), 7.36–7.45 (*m*, 2H, Ar-H), 7.47–7.50 (*m*, 1H, Ar-H), 7.61–7.67 (*m*, 2H, Ar-H), 7.83–7.95 (*m*, 3H, Ar-H), 8.12 (*s*, 1H), 8.13 (*s*, 1H), 11.64 (*s*, 1H). ^13^C NMR (400 MHz, DMSO-*d*_6_, *δ* ppm): 43.27, 44.49, 126.82, 127.53, 128.54, 129.11, 129.82, 131.25, 133.02, 137.24, 139.19, 139.81, 143.18, 164.23, 169.08.

**Compound (II):** Prism-like colourless single crystals; m.p. 469–470 K; IR (KBr, γ, cm^−1^): 3265.5 (N—-H), 1687.7 (C=O), 1589.3 (C=N), 1340.5 (S=O, asym) and 1168.9 cm^−1^ (S=O, sym); ^1^H NMR (400 MHz, DMSO-*d*_6_, *δ* ppm): 3.64, 4.11 (2*d*, 2H), 7.39–7.44 (*m*, 2H, Ar-H), 7.54–7.65 (*m*, 4H, Ar-H), 7.84–7.87 (*m*, 2H, Ar-H), 7.91, 8.15 (2*s*, 1H), 8.01, 8.21 (2*t*, 1H), 11.51, 11.54 (2*s*, 1H). ^13^C NMR (400 MHz, DMSO-*d*_6_, *δ* ppm): 43.24, 44.42, 125.52, 126.14, 128.44, 128.91, 129.47, 130.31, 133.70, 136.05, 137.34, 139.36, 142.18, 145.60, 164.17, 168.96.

**Compound (III):** Rod-like colourless single crystals; m.p. 473–475 K; IR (KBr, γ, cm^−1^): 3246.2 (N—H), 1685.8 (C=O), 1591.3 (C=N), 1344.4 (S=O, asym) and 1168.9 cm^−1^ (S=O, sym); ^1^H NMR (400 MHz, DMSO-*d*_6_, *δ* ppm): 3.62, 4.11 (2*d*, 2H), 7.48–7.51 (*m*, 2H, Ar-H), 7.63–7.71 (*m*, 4H, Ar-H), 7.81–7.85 (*m*, 2H, Ar-H), 7.92, 8.14 (2*s*, 1H), 8.01 (*t*, 1H), 11.49, 11.53 (2*s*, 1H). ^13^C NMR (400 MHz, DMSO-*d*_6_, *δ* ppm): 43.21, 128.50, 128.70, 128.86, 129.14, 132.87, 134.34, 137.20, 139.50, 142.53, 145.80, 164.07, 168.96.

## Refinement details   

Crystal data, data collection and structure refinement details are summarized in Table 4 ▸. H atoms bonded to C were positioned with idealized geometry using a riding model with C—H = 0.93 Å (aromatic) and 0.97 Å (methyl­ene). The amino H atoms were refined with the N—H distances restrained to 0.86 (2) Å. All H atoms were assigned isotropic displacement parameters 1.2 × *U*_eq_ of the parent atom. In compound (III), the 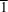 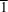 3 reflection had a poor agreement with its calculated value and was omitted from the final refinement.

## Supplementary Material

Crystal structure: contains datablock(s) I, II, III, global. DOI: 10.1107/S2056989021006885/mw2177sup1.cif

Structure factors: contains datablock(s) I. DOI: 10.1107/S2056989021006885/mw2177Isup2.hkl

Structure factors: contains datablock(s) II. DOI: 10.1107/S2056989021006885/mw2177IIsup3.hkl

Structure factors: contains datablock(s) III. DOI: 10.1107/S2056989021006885/mw2177IIIsup4.hkl

Supporting information file. DOI: 10.1107/S2056989021006885/mw2177Isup5.cml

Supporting information file. DOI: 10.1107/S2056989021006885/mw2177IIsup6.cml

CCDC references: 1433593, 1433594, 1433606

Additional supporting information:  crystallographic information; 3D view; checkCIF report

## Figures and Tables

**Figure 2:**
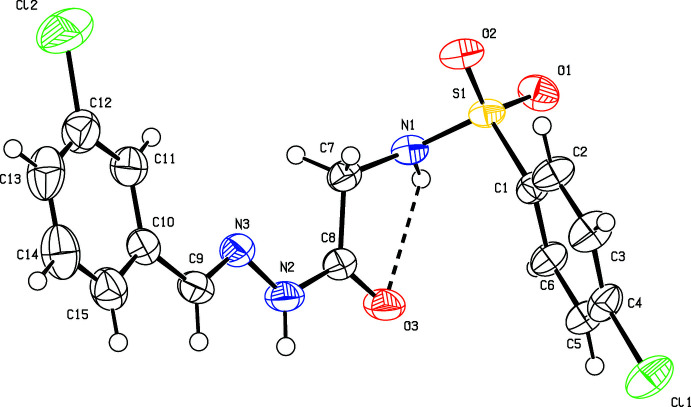
The mol­ecular structure of (II), showing the atom labelling and displacement ellipsoids drawn at the 50% probability level. The intra­molecular hydrogen bond is depicted by a dashed line.

**Figure 3:**
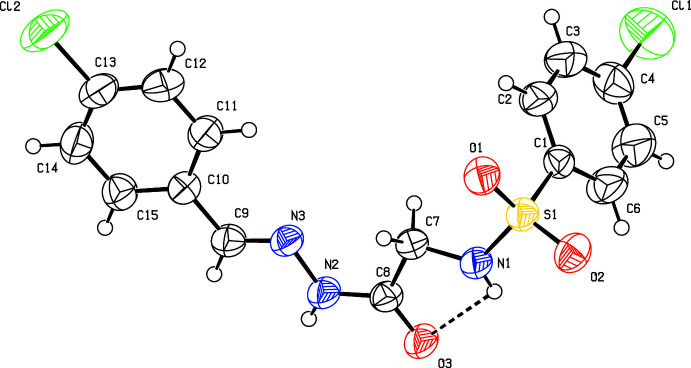
The mol­ecular structure of (III), showing the atom labelling and displacement ellipsoids drawn at the 50% probability level. The intra­molecular hydrogen bond is depicted by a dashed line.

**Table 1:** Hydrogen-bond geometry (Å, °) for (I)

*D*—H⋯*A*	*D*—H	H⋯*A*	*D*⋯*A*	*D*—H⋯*A*
N1—H1*N*⋯O3^i^	0.84 (2)	2.01 (2)	2.823 (3)	162 (3)
N2—H2*N*⋯O3^ii^	0.85 (2)	2.06 (2)	2.897 (2)	167 (2)
C12—H12⋯O2^iii^	0.93	2.53	3.439 (3)	166

Symmetry codes: (i) -x+1, -y+1, -z+1; (ii) -x+2, -y+1, -z+1; (iii) x+1, y+1, z.

**Table 2:** Hydrogen-bond geometry (Å, °) for (II) *Cg* is the centroid of the C1–C6 ring.

*D*—H⋯*A*	*D*—H	H⋯*A*	*D*⋯*A*	*D*—H⋯*A*
N1—H1*N*⋯O1^i^	0.84 (2)	2.23 (2)	3.041 (3)	161 (3)
N2—H2*N*⋯O3^ii^	0.86 (2)	1.97 (2)	2.828 (3)	171 (3)
C6—H6⋯O1^i^	0.93	2.53	3.422 (4)	161
C7—H7*B*⋯O2^iii^	0.97	2.47	3.434 (4)	172
C15—H15⋯O2^iv^	0.93	2.58	3.474 (4)	162
C14—H14⋯*Cg* ^v^	0.93	2.84	3.675 (5)	150

Symmetry codes: (i) -x+1, -y, -z+2; (ii) -x+1, -y, -z+1; (iii) -x, -y, -z+2; (iv) x, y, z-1; (v) -x, -y, -z+1.

**Table 3:** Hydrogen-bond geometry (Å, °) for (III)

*D*—H⋯*A*	*D*—H	H⋯*A*	*D*⋯*A*	*D*—H⋯*A*
N1—H1*N*⋯O2^i^	0.82 (2)	2.25 (2)	3.032 (3)	162 (3)
N1—H1*N*⋯O3	0.82 (2)	2.23 (3)	2.609 (3)	109 (2)
N2—H2*N*⋯O3^ii^	0.86 (2)	1.98 (2)	2.829 (3)	170 (3)
C15—H15⋯O1^iii^	0.93	2.45	3.330 (3)	157

Symmetry codes: (i) -x-1, -y+2, -z; (ii) -x, -y+1, -z; (iii) x+1, y-1, z.

**Table 4:** Experimental details

	(I)	(II)	(III)
Crystal data			
Chemical formula	C_15_H_13_Cl_2_N_3_O_3_S	C_15_H_13_Cl_2_N_3_O_3_S	C_15_H_13_Cl_2_N_3_O_3_S
*M*_r_	386.24	386.24	386.24
Crystal system, space group	Triclinic, *P*\overline{1}	Triclinic, *P*\overline{1}	Triclinic, *P*\overline{1}
Temperature (K)	293	293	293
*a*, *b*, *c* (Å)	7.7426 (7), 10.429 (1), 10.934 (1)	9.491 (1), 9.976 (1), 10.446 (1)	6.7234 (9), 10.281 (1), 13.611 (2)
α, β, γ (°)	85.51 (1), 76.92 (1), 81.04 (1)	67.22 (1), 66.80 (1), 86.32 (1)	74.98 (1), 87.11 (1), 75.34 (1)
*V* (Å^3^)	848.64 (14)	833.59 (17)	879.0 (2)
*Z*	2	2	2
Radiation type	Mo *K*α	Mo *K*α	Mo *K*α
μ (mm^−1^)	0.52	0.53	0.51
Crystal size (mm)	0.48 × 0.36 × 0.10	0.36 × 0.14 × 0.08	0.46 × 0.42 × 0.20

Data collection			
Diffractometer	Oxford Diffraction Xcalibur with Sapphire CCD	Oxford Diffraction Xcalibur with Sapphire CCD	Oxford Diffraction Xcalibur with Sapphire CCD
Absorption correction	Multi-scan (*CrysAlis RED*; Oxford Diffraction, 2009 ▸)	Multi-scan (*CrysAlis RED*, Oxford Diffraction, 2009 ▸)	Multi-scan (*CrysAlis RED*, Oxford Diffraction, 2009 ▸)
*T*_min_, *T*_max_	0.787, 0.949	0.831, 0.959	0.801, 0.906
No. of measured, independent and observed [*I* > 2σ(*I*)] reflections	5699, 3417, 2359	5759, 3354, 2688	5737, 3598, 2600
*R*_int_	0.018	0.018	0.019
(sin θ/λ)_max_ (Å^−1^)	0.625	0.625	0.625

Refinement			
*R*[*F* ^2^ > 2σ(*F* ^2^)], *wR*(*F* ^2^), *S*	0.046, 0.102, 1.04	0.054, 0.108, 1.22	0.048, 0.120, 1.04
No. of reflections	3417	3354	3598
No. of parameters	223	223	223
No. of restraints	2	2	2
H-atom treatment	H atoms treated by a mixture of independent and constrained refinement	H atoms treated by a mixture of independent and constrained refinement	H atoms treated by a mixture of independent and constrained refinement
Δρ_max_, Δρ_min_ (e Å^−3^)	0.24, −0.29	0.27, −0.39	0.33, −0.30

Computer programs: *CrysAlis CCD* and *CrysAlis RED* (Oxford Diffraction, 2009 ▸), *SHELXS2013/1* (Sheldrick, 2008 ▸), *SHELXL2014/6* (Sheldrick, 2015 ▸) and *PLATON* (Spek, 2020 ▸).

## supplementary crystallographic information

### (E)-4-Chloro-N-{2-[2-(2-chlorobenzylidene)hydrazinyl]-2-oxoethyl}benzenesulfonamide (I) Crystal data

**Table:**

C_15_H_13_Cl_2_N_3_O_3_S	*Z* = 2
*M**_r_* = 386.24	*F*(000) = 396
Triclinic, *P*1	*D*_x_ = 1.512 Mg m^−^^3^
*a* = 7.7426 (7) Å	Mo *K*α radiation, λ = 0.71073 Å
*b* = 10.429 (1) Å	Cell parameters from 2012 reflections
*c* = 10.934 (1) Å	θ = 2.8–27.8°
α = 85.51 (1)°	µ = 0.52 mm^−^^1^
β = 76.92 (1)°	*T* = 293 K
γ = 81.04 (1)°	Prism, yellow
*V* = 848.64 (14) Å^3^	0.48 × 0.36 × 0.10 mm

### (E)-4-Chloro-N-{2-[2-(2-chlorobenzylidene)hydrazinyl]-2-oxoethyl}benzenesulfonamide (I) Data collection

**Table:**

Oxford Diffraction Xcalibur with Sapphire CCD diffractometer	2359 reflections with *I* > 2σ(*I*)
Radiation source: Enhance (Mo) X-ray Source	*R*_int_ = 0.018
Rotation method data acquisition using ω scans.	θ_max_ = 26.4°, θ_min_ = 2.8°
Absorption correction: multi-scan (CrysAlis RED; Oxford Diffraction, 2009)	*h* = −9→9
*T*_min_ = 0.787, *T*_max_ = 0.949	*k* = −10→13
5699 measured reflections	*l* = −13→13
3417 independent reflections	

### (E)-4-Chloro-N-{2-[2-(2-chlorobenzylidene)hydrazinyl]-2-oxoethyl}benzenesulfonamide (I) Refinement

**Table:**

Refinement on *F*^2^	2 restraints
Least-squares matrix: full	Hydrogen site location: mixed
*R*[*F*^2^ > 2σ(*F*^2^)] = 0.046	H atoms treated by a mixture of independent and constrained refinement
*wR*(*F*^2^) = 0.102	*w* = 1/[σ^2^(*F*_o_^2^) + (0.0321*P*)^2^ + 0.3938*P*] where *P* = (*F*_o_^2^ + 2*F*_c_^2^)/3
*S* = 1.04	(Δ/σ)_max_ = 0.001
3417 reflections	Δρ_max_ = 0.24 e Å^−^^3^
223 parameters	Δρ_min_ = −0.29 e Å^−^^3^

### (E)-4-Chloro-N-{2-[2-(2-chlorobenzylidene)hydrazinyl]-2-oxoethyl}benzenesulfonamide (I) Special details

**Table:**

Experimental. CrysAlis RED (Oxford Diffraction, 2009) Empirical absorption correction using spherical harmonics, implemented in SCALE3 ABSPACK scaling algorithm.
Geometry. All esds (except the esd in the dihedral angle between two l.s. planes) are estimated using the full covariance matrix. The cell esds are taken into account individually in the estimation of esds in distances, angles and torsion angles; correlations between esds in cell parameters are only used when they are defined by crystal symmetry. An approximate (isotropic) treatment of cell esds is used for estimating esds involving l.s. planes.

### (E)-4-Chloro-N-{2-[2-(2-chlorobenzylidene)hydrazinyl]-2-oxoethyl}benzenesulfonamide (I) Fractional atomic coordinates and isotropic or equivalent isotropic displacement parameters (Å^2^)

**Table:**

	*x*	*y*	*z*	*U*_iso_*/*U*_eq_	
C1	0.1746 (3)	0.6076 (2)	0.8440 (2)	0.0487 (6)	
C2	0.1897 (4)	0.7192 (3)	0.8985 (3)	0.0687 (8)	
H2	0.3023	0.7399	0.8985	0.082*	
C3	0.0378 (5)	0.7997 (3)	0.9529 (3)	0.0852 (11)	
H3	0.0473	0.8756	0.9888	0.102*	
C4	−0.1270 (5)	0.7675 (4)	0.9540 (3)	0.0801 (11)	
C5	−0.1448 (4)	0.6551 (4)	0.9042 (3)	0.0763 (10)	
H5	−0.2579	0.6330	0.9083	0.092*	
C6	0.0079 (4)	0.5742 (3)	0.8473 (3)	0.0595 (7)	
H6	−0.0022	0.4982	0.8118	0.071*	
C7	0.5597 (3)	0.6727 (2)	0.6046 (3)	0.0521 (7)	
H7A	0.5504	0.7198	0.6794	0.063*	
H7B	0.5376	0.7357	0.5378	0.063*	
C8	0.7483 (3)	0.6002 (2)	0.5670 (2)	0.0386 (5)	
C9	0.9738 (3)	0.8484 (2)	0.6184 (2)	0.0405 (6)	
H9	1.0910	0.8074	0.5943	0.049*	
C10	0.9368 (3)	0.9816 (2)	0.6622 (2)	0.0381 (5)	
C11	1.0705 (3)	1.0515 (2)	0.6753 (2)	0.0430 (6)	
C12	1.0322 (4)	1.1777 (2)	0.7149 (3)	0.0545 (7)	
H12	1.1239	1.2217	0.7240	0.065*	
C13	0.8572 (4)	1.2374 (3)	0.7408 (3)	0.0616 (8)	
H13	0.8306	1.3224	0.7669	0.074*	
C14	0.7213 (4)	1.1717 (3)	0.7281 (3)	0.0614 (8)	
H14	0.6032	1.2124	0.7452	0.074*	
C15	0.7610 (3)	1.0457 (2)	0.6902 (3)	0.0516 (7)	
H15	0.6681	1.0019	0.6831	0.062*	
N1	0.4241 (3)	0.5872 (2)	0.6292 (2)	0.0526 (6)	
H1N	0.383 (3)	0.569 (3)	0.569 (2)	0.063*	
N2	0.8820 (3)	0.66560 (18)	0.5716 (2)	0.0420 (5)	
H2N	0.990 (2)	0.631 (2)	0.544 (2)	0.050*	
N3	0.8430 (2)	0.79018 (18)	0.61392 (19)	0.0411 (5)	
O1	0.5090 (3)	0.5131 (2)	0.8282 (2)	0.0751 (6)	
O2	0.3180 (3)	0.39150 (17)	0.7388 (2)	0.0732 (6)	
O3	0.7778 (2)	0.48784 (15)	0.53116 (17)	0.0483 (4)	
Cl1	−0.31736 (16)	0.87209 (13)	1.01893 (11)	0.1407 (6)	
Cl2	1.29502 (9)	0.98193 (7)	0.63926 (8)	0.0690 (3)	
S1	0.36939 (9)	0.51159 (7)	0.76264 (7)	0.0529 (2)	

### (E)-4-Chloro-N-{2-[2-(2-chlorobenzylidene)hydrazinyl]-2-oxoethyl}benzenesulfonamide (I) Atomic displacement parameters (Å^2^)

**Table:**

	*U*^11^	*U*^22^	*U*^33^	*U*^12^	*U*^13^	*U*^23^
C1	0.0476 (15)	0.0454 (15)	0.0497 (16)	−0.0040 (12)	−0.0062 (12)	0.0005 (12)
C2	0.0685 (19)	0.062 (2)	0.071 (2)	−0.0128 (16)	−0.0002 (16)	−0.0158 (17)
C3	0.105 (3)	0.062 (2)	0.071 (2)	0.004 (2)	0.011 (2)	−0.0138 (18)
C4	0.079 (2)	0.077 (2)	0.056 (2)	0.026 (2)	0.0122 (17)	0.0121 (18)
C5	0.0474 (17)	0.105 (3)	0.064 (2)	0.0034 (18)	−0.0040 (15)	0.019 (2)
C6	0.0504 (16)	0.0663 (19)	0.0597 (18)	−0.0082 (14)	−0.0104 (14)	0.0043 (15)
C7	0.0380 (13)	0.0404 (15)	0.0759 (19)	−0.0050 (11)	−0.0040 (13)	−0.0151 (13)
C8	0.0374 (12)	0.0324 (13)	0.0450 (14)	−0.0041 (10)	−0.0076 (11)	−0.0029 (11)
C9	0.0360 (12)	0.0333 (13)	0.0513 (15)	−0.0035 (10)	−0.0079 (11)	−0.0032 (11)
C10	0.0392 (12)	0.0289 (12)	0.0452 (14)	−0.0035 (10)	−0.0077 (11)	−0.0018 (11)
C11	0.0402 (13)	0.0371 (13)	0.0525 (16)	−0.0063 (10)	−0.0113 (11)	−0.0021 (12)
C12	0.0604 (17)	0.0397 (15)	0.0690 (19)	−0.0164 (13)	−0.0179 (14)	−0.0070 (14)
C13	0.075 (2)	0.0322 (14)	0.077 (2)	−0.0029 (14)	−0.0153 (16)	−0.0124 (14)
C14	0.0508 (16)	0.0414 (16)	0.086 (2)	0.0051 (13)	−0.0068 (15)	−0.0124 (15)
C15	0.0414 (14)	0.0391 (14)	0.0737 (19)	−0.0058 (11)	−0.0096 (13)	−0.0072 (13)
N1	0.0412 (12)	0.0617 (15)	0.0576 (15)	−0.0181 (11)	−0.0039 (10)	−0.0171 (12)
N2	0.0340 (10)	0.0305 (11)	0.0600 (14)	−0.0033 (9)	−0.0053 (10)	−0.0093 (10)
N3	0.0405 (11)	0.0298 (10)	0.0529 (13)	−0.0052 (9)	−0.0086 (9)	−0.0057 (9)
O1	0.0577 (12)	0.0822 (15)	0.0895 (16)	0.0029 (11)	−0.0313 (11)	−0.0095 (13)
O2	0.0696 (13)	0.0384 (11)	0.1099 (18)	−0.0079 (10)	−0.0132 (12)	−0.0116 (11)
O3	0.0393 (9)	0.0342 (9)	0.0725 (12)	−0.0030 (7)	−0.0115 (8)	−0.0153 (9)
Cl1	0.1177 (9)	0.1400 (10)	0.1047 (8)	0.0695 (8)	0.0332 (7)	0.0151 (7)
Cl2	0.0391 (4)	0.0617 (5)	0.1092 (7)	−0.0066 (3)	−0.0181 (4)	−0.0182 (4)
S1	0.0428 (4)	0.0433 (4)	0.0722 (5)	−0.0029 (3)	−0.0113 (3)	−0.0093 (3)

### (E)-4-Chloro-N-{2-[2-(2-chlorobenzylidene)hydrazinyl]-2-oxoethyl}benzenesulfonamide (I) Geometric parameters (Å, º)

**Table:**

C1—C2	1.380 (4)	C9—C10	1.469 (3)
C1—C6	1.381 (4)	C9—H9	0.9300
C1—S1	1.769 (3)	C10—C11	1.394 (3)
C2—C3	1.377 (4)	C10—C15	1.398 (3)
C2—H2	0.9300	C11—C12	1.385 (3)
C3—C4	1.367 (5)	C11—Cl2	1.745 (2)
C3—H3	0.9300	C12—C13	1.376 (4)
C4—C5	1.369 (5)	C12—H12	0.9300
C4—Cl1	1.736 (3)	C13—C14	1.379 (4)
C5—C6	1.391 (4)	C13—H13	0.9300
C5—H5	0.9300	C14—C15	1.377 (3)
C6—H6	0.9300	C14—H14	0.9300
C7—N1	1.447 (3)	C15—H15	0.9300
C7—C8	1.518 (3)	N1—S1	1.604 (2)
C7—H7A	0.9700	N1—H1N	0.841 (16)
C7—H7B	0.9700	N2—N3	1.379 (3)
C8—O3	1.236 (3)	N2—H2N	0.852 (16)
C8—N2	1.337 (3)	O1—S1	1.4283 (19)
C9—N3	1.271 (3)	O2—S1	1.4312 (19)
			
C2—C1—C6	120.3 (3)	C11—C10—C9	123.2 (2)
C2—C1—S1	119.8 (2)	C15—C10—C9	120.2 (2)
C6—C1—S1	119.8 (2)	C12—C11—C10	122.1 (2)
C3—C2—C1	119.9 (3)	C12—C11—Cl2	117.61 (19)
C3—C2—H2	120.1	C10—C11—Cl2	120.32 (18)
C1—C2—H2	120.1	C13—C12—C11	119.4 (2)
C4—C3—C2	119.6 (3)	C13—C12—H12	120.3
C4—C3—H3	120.2	C11—C12—H12	120.3
C2—C3—H3	120.2	C12—C13—C14	120.2 (2)
C3—C4—C5	121.4 (3)	C12—C13—H13	119.9
C3—C4—Cl1	119.1 (3)	C14—C13—H13	119.9
C5—C4—Cl1	119.5 (3)	C15—C14—C13	119.7 (3)
C4—C5—C6	119.4 (3)	C15—C14—H14	120.1
C4—C5—H5	120.3	C13—C14—H14	120.1
C6—C5—H5	120.3	C14—C15—C10	121.9 (2)
C1—C6—C5	119.4 (3)	C14—C15—H15	119.0
C1—C6—H6	120.3	C10—C15—H15	119.0
C5—C6—H6	120.3	C7—N1—S1	122.21 (19)
N1—C7—C8	112.8 (2)	C7—N1—H1N	119 (2)
N1—C7—H7A	109.0	S1—N1—H1N	119 (2)
C8—C7—H7A	109.0	C8—N2—N3	119.52 (19)
N1—C7—H7B	109.0	C8—N2—H2N	119.0 (17)
C8—C7—H7B	109.0	N3—N2—H2N	121.4 (17)
H7A—C7—H7B	107.8	C9—N3—N2	117.61 (19)
O3—C8—N2	121.5 (2)	O1—S1—O2	120.84 (13)
O3—C8—C7	122.1 (2)	O1—S1—N1	107.43 (13)
N2—C8—C7	116.5 (2)	O2—S1—N1	107.08 (12)
N3—C9—C10	119.0 (2)	O1—S1—C1	107.99 (13)
N3—C9—H9	120.5	O2—S1—C1	107.31 (12)
C10—C9—H9	120.5	N1—S1—C1	105.16 (12)
C11—C10—C15	116.6 (2)		
			
C6—C1—C2—C3	−2.1 (4)	C11—C12—C13—C14	0.5 (4)
S1—C1—C2—C3	174.7 (2)	C12—C13—C14—C15	0.4 (5)
C1—C2—C3—C4	0.9 (5)	C13—C14—C15—C10	−0.8 (4)
C2—C3—C4—C5	1.3 (5)	C11—C10—C15—C14	0.4 (4)
C2—C3—C4—Cl1	−178.2 (2)	C9—C10—C15—C14	−178.1 (3)
C3—C4—C5—C6	−2.3 (5)	C8—C7—N1—S1	83.6 (3)
Cl1—C4—C5—C6	177.2 (2)	O3—C8—N2—N3	−179.0 (2)
C2—C1—C6—C5	1.1 (4)	C7—C8—N2—N3	2.6 (3)
S1—C1—C6—C5	−175.7 (2)	C10—C9—N3—N2	179.6 (2)
C4—C5—C6—C1	1.1 (4)	C8—N2—N3—C9	179.1 (2)
N1—C7—C8—O3	17.0 (4)	C7—N1—S1—O1	−17.3 (2)
N1—C7—C8—N2	−164.6 (2)	C7—N1—S1—O2	−148.5 (2)
N3—C9—C10—C11	177.3 (2)	C7—N1—S1—C1	97.6 (2)
N3—C9—C10—C15	−4.3 (4)	C2—C1—S1—O1	36.9 (3)
C15—C10—C11—C12	0.5 (4)	C6—C1—S1—O1	−146.3 (2)
C9—C10—C11—C12	179.0 (2)	C2—C1—S1—O2	168.7 (2)
C15—C10—C11—Cl2	−178.54 (19)	C6—C1—S1—O2	−14.6 (3)
C9—C10—C11—Cl2	0.0 (3)	C2—C1—S1—N1	−77.6 (2)
C10—C11—C12—C13	−0.9 (4)	C6—C1—S1—N1	99.2 (2)
Cl2—C11—C12—C13	178.1 (2)		

### (E)-4-Chloro-N-{2-[2-(2-chlorobenzylidene)hydrazinyl]-2-oxoethyl}benzenesulfonamide (I) Hydrogen-bond geometry (Å, º)

**Table:**

*D*—H···*A*	*D*—H	H···*A*	*D*···*A*	*D*—H···*A*
N1—H1*N*···O3^i^	0.84 (2)	2.01 (2)	2.823 (3)	162 (3)
N2—H2*N*···O3^ii^	0.85 (2)	2.06 (2)	2.897 (2)	167 (2)
C12—H12···O2^iii^	0.93	2.53	3.439 (3)	166

Symmetry codes: (i) −*x*+1, −*y*+1, −*z*+1; (ii) −*x*+2, −*y*+1, −*z*+1; (iii) *x*+1, *y*+1, *z*.

### (E)-4-Chloro-N-{2-[2-(3-chlorobenzylidene)hydrazinyl]-2-oxoethyl}benzenesulfonamide (II) Crystal data

**Table:**

C_15_H_13_Cl_2_N_3_O_3_S	*Z* = 2
*M**_r_* = 386.24	*F*(000) = 396
Triclinic, *P*1	*D*_x_ = 1.539 Mg m^−^^3^
*a* = 9.491 (1) Å	Mo *K*α radiation, λ = 0.71073 Å
*b* = 9.976 (1) Å	Cell parameters from 2850 reflections
*c* = 10.446 (1) Å	θ = 2.6–27.9°
α = 67.22 (1)°	µ = 0.53 mm^−^^1^
β = 66.80 (1)°	*T* = 293 K
γ = 86.32 (1)°	Prism, colourless
*V* = 833.59 (17) Å^3^	0.36 × 0.14 × 0.08 mm

### (E)-4-Chloro-N-{2-[2-(3-chlorobenzylidene)hydrazinyl]-2-oxoethyl}benzenesulfonamide (II) Data collection

**Table:**

Oxford Diffraction Xcalibur with Sapphire CCD diffractometer	2688 reflections with *I* > 2σ(*I*)
Radiation source: fine-focus sealed tube	*R*_int_ = 0.018
Rotation method data acquisition using ω scans.	θ_max_ = 26.4°, θ_min_ = 2.6°
Absorption correction: multi-scan (CrysAlis RED, Oxford Diffraction, 2009)	*h* = −11→11
*T*_min_ = 0.831, *T*_max_ = 0.959	*k* = −12→12
5759 measured reflections	*l* = −13→12
3354 independent reflections	

### (E)-4-Chloro-N-{2-[2-(3-chlorobenzylidene)hydrazinyl]-2-oxoethyl}benzenesulfonamide (II) Refinement

**Table:**

Refinement on *F*^2^	Primary atom site location: structure-invariant direct methods
Least-squares matrix: full	Secondary atom site location: difference Fourier map
*R*[*F*^2^ > 2σ(*F*^2^)] = 0.054	Hydrogen site location: mixed
*wR*(*F*^2^) = 0.108	H atoms treated by a mixture of independent and constrained refinement
*S* = 1.22	*w* = 1/[σ^2^(*F*_o_^2^) + (0.0148*P*)^2^ + 1.1114*P*] where *P* = (*F*_o_^2^ + 2*F*_c_^2^)/3
3354 reflections	(Δ/σ)_max_ = 0.002
223 parameters	Δρ_max_ = 0.27 e Å^−^^3^
2 restraints	Δρ_min_ = −0.39 e Å^−^^3^

### (E)-4-Chloro-N-{2-[2-(3-chlorobenzylidene)hydrazinyl]-2-oxoethyl}benzenesulfonamide (II) Special details

**Table:**

Experimental. CrysAlis RED, Oxford Diffraction Ltd., 2009 Empirical absorption correction using spherical harmonics, implemented in SCALE3 ABSPACK scaling algorithm.
Geometry. All esds (except the esd in the dihedral angle between two l.s. planes) are estimated using the full covariance matrix. The cell esds are taken into account individually in the estimation of esds in distances, angles and torsion angles; correlations between esds in cell parameters are only used when they are defined by crystal symmetry. An approximate (isotropic) treatment of cell esds is used for estimating esds involving l.s. planes.
Refinement. Refinement of F^2^ against ALL reflections. The weighted R-factor wR and goodness of fit S are based on F^2^, conventional R-factors R are based on F, with F set to zero for negative F^2^. The threshold expression of F^2^ > 2sigma(F^2^) is used only for calculating R-factors(gt) etc. and is not relevant to the choice of reflections for refinement. R-factors based on F^2^ are statistically about twice as large as those based on F, and R- factors based on ALL data will be even larger.

### (E)-4-Chloro-N-{2-[2-(3-chlorobenzylidene)hydrazinyl]-2-oxoethyl}benzenesulfonamide (II) Fractional atomic coordinates and isotropic or equivalent isotropic displacement parameters (Å^2^)

**Table:**

	*x*	*y*	*z*	*U*_iso_*/*U*_eq_	
C1	0.2520 (3)	−0.1930 (3)	1.0295 (3)	0.0312 (6)	
C2	0.1178 (3)	−0.2828 (3)	1.0900 (4)	0.0408 (7)	
H2	0.0241	−0.2549	1.1415	0.049*	
C3	0.1226 (4)	−0.4145 (4)	1.0739 (4)	0.0458 (8)	
H3	0.0329	−0.4769	1.1166	0.055*	
C4	0.2616 (4)	−0.4519 (3)	0.9939 (4)	0.0392 (7)	
C5	0.3966 (4)	−0.3626 (3)	0.9327 (4)	0.0393 (7)	
H5	0.4898	−0.3896	0.8789	0.047*	
C6	0.3924 (3)	−0.2326 (3)	0.9519 (3)	0.0356 (7)	
H6	0.4828	−0.1724	0.9131	0.043*	
C7	0.2808 (3)	0.1427 (3)	0.7715 (3)	0.0356 (7)	
H7A	0.2814	0.2480	0.7273	0.043*	
H7B	0.1751	0.0995	0.8162	0.043*	
C8	0.3771 (3)	0.0903 (3)	0.6490 (3)	0.0351 (7)	
C9	0.1510 (4)	0.1675 (4)	0.4412 (3)	0.0414 (7)	
H9	0.2108	0.1262	0.3740	0.050*	
C10	0.0084 (4)	0.2250 (3)	0.4311 (3)	0.0400 (7)	
C11	−0.0769 (4)	0.3041 (4)	0.5152 (4)	0.0469 (8)	
H11	−0.0426	0.3245	0.5783	0.056*	
C12	−0.2121 (4)	0.3515 (4)	0.5037 (4)	0.0540 (9)	
C13	−0.2654 (4)	0.3247 (4)	0.4096 (5)	0.0600 (10)	
H13	−0.3566	0.3589	0.4022	0.072*	
C14	−0.1815 (5)	0.2467 (4)	0.3273 (5)	0.0603 (10)	
H14	−0.2165	0.2274	0.2640	0.072*	
C15	−0.0459 (4)	0.1966 (4)	0.3371 (4)	0.0497 (8)	
H15	0.0097	0.1435	0.2808	0.060*	
Cl1	0.26935 (11)	−0.61454 (10)	0.96808 (12)	0.0584 (3)	
Cl2	−0.32203 (15)	0.44557 (16)	0.61290 (17)	0.0976 (5)	
N1	0.3402 (3)	0.1031 (3)	0.8891 (3)	0.0318 (5)	
H1N	0.436 (2)	0.101 (3)	0.860 (3)	0.038*	
N2	0.3309 (3)	0.1124 (3)	0.5371 (3)	0.0436 (7)	
H2N	0.378 (4)	0.072 (4)	0.474 (3)	0.052*	
N3	0.1952 (3)	0.1727 (3)	0.5398 (3)	0.0394 (6)	
O1	0.3340 (2)	−0.0350 (2)	1.1413 (2)	0.0411 (5)	
O2	0.0898 (2)	0.0034 (2)	1.1055 (2)	0.0408 (5)	
O3	0.4936 (2)	0.0311 (3)	0.6515 (2)	0.0475 (6)	
S1	0.24739 (8)	−0.02589 (8)	1.05320 (8)	0.03071 (18)	

### (E)-4-Chloro-N-{2-[2-(3-chlorobenzylidene)hydrazinyl]-2-oxoethyl}benzenesulfonamide (II) Atomic displacement parameters (Å^2^)

**Table:**

	*U*^11^	*U*^22^	*U*^33^	*U*^12^	*U*^13^	*U*^23^
C1	0.0333 (15)	0.0300 (15)	0.0385 (16)	0.0073 (12)	−0.0189 (13)	−0.0176 (13)
C2	0.0280 (16)	0.0415 (18)	0.057 (2)	0.0072 (13)	−0.0145 (15)	−0.0268 (16)
C3	0.0347 (17)	0.0386 (18)	0.069 (2)	0.0006 (14)	−0.0209 (17)	−0.0261 (17)
C4	0.0461 (19)	0.0299 (16)	0.0530 (19)	0.0096 (14)	−0.0279 (16)	−0.0204 (15)
C5	0.0347 (17)	0.0377 (17)	0.0496 (19)	0.0124 (13)	−0.0173 (15)	−0.0224 (15)
C6	0.0291 (15)	0.0343 (16)	0.0469 (18)	0.0061 (12)	−0.0159 (14)	−0.0192 (14)
C7	0.0354 (16)	0.0374 (17)	0.0375 (16)	0.0116 (13)	−0.0156 (14)	−0.0185 (14)
C8	0.0341 (16)	0.0371 (17)	0.0339 (16)	0.0086 (13)	−0.0129 (13)	−0.0153 (14)
C9	0.0435 (18)	0.050 (2)	0.0326 (16)	0.0112 (15)	−0.0145 (14)	−0.0195 (15)
C10	0.0422 (18)	0.0414 (18)	0.0329 (16)	0.0052 (14)	−0.0150 (14)	−0.0113 (14)
C11	0.047 (2)	0.054 (2)	0.0451 (19)	0.0126 (16)	−0.0246 (16)	−0.0199 (17)
C12	0.048 (2)	0.052 (2)	0.052 (2)	0.0109 (17)	−0.0181 (18)	−0.0131 (18)
C13	0.044 (2)	0.060 (2)	0.067 (3)	0.0048 (18)	−0.031 (2)	−0.006 (2)
C14	0.065 (3)	0.060 (2)	0.062 (2)	−0.004 (2)	−0.042 (2)	−0.011 (2)
C15	0.062 (2)	0.047 (2)	0.0440 (19)	0.0028 (17)	−0.0269 (18)	−0.0156 (16)
Cl1	0.0641 (6)	0.0405 (5)	0.0897 (7)	0.0132 (4)	−0.0367 (5)	−0.0401 (5)
Cl2	0.0847 (9)	0.1137 (11)	0.1072 (10)	0.0590 (8)	−0.0404 (8)	−0.0618 (9)
N1	0.0274 (12)	0.0354 (13)	0.0368 (14)	0.0033 (11)	−0.0109 (11)	−0.0205 (11)
N2	0.0416 (16)	0.0597 (18)	0.0408 (15)	0.0224 (13)	−0.0198 (13)	−0.0303 (14)
N3	0.0378 (14)	0.0457 (16)	0.0368 (14)	0.0134 (12)	−0.0165 (12)	−0.0183 (12)
O1	0.0400 (12)	0.0535 (14)	0.0406 (12)	0.0020 (10)	−0.0200 (10)	−0.0249 (11)
O2	0.0309 (11)	0.0474 (13)	0.0512 (13)	0.0071 (9)	−0.0115 (10)	−0.0320 (11)
O3	0.0404 (13)	0.0727 (16)	0.0418 (13)	0.0248 (12)	−0.0208 (11)	−0.0336 (12)
S1	0.0279 (4)	0.0352 (4)	0.0363 (4)	0.0050 (3)	−0.0127 (3)	−0.0219 (3)

### (E)-4-Chloro-N-{2-[2-(3-chlorobenzylidene)hydrazinyl]-2-oxoethyl}benzenesulfonamide (II) Geometric parameters (Å, º)

**Table:**

C1—C2	1.377 (4)	C9—C10	1.462 (4)
C1—C6	1.385 (4)	C9—H9	0.9300
C1—S1	1.773 (3)	C10—C15	1.394 (4)
C2—C3	1.384 (4)	C10—C11	1.394 (4)
C2—H2	0.9300	C11—C12	1.372 (5)
C3—C4	1.374 (4)	C11—H11	0.9300
C3—H3	0.9300	C12—C13	1.381 (5)
C4—C5	1.380 (4)	C12—Cl2	1.743 (4)
C4—Cl1	1.737 (3)	C13—C14	1.370 (6)
C5—C6	1.382 (4)	C13—H13	0.9300
C5—H5	0.9300	C14—C15	1.378 (5)
C6—H6	0.9300	C14—H14	0.9300
C7—N1	1.457 (4)	C15—H15	0.9300
C7—C8	1.510 (4)	N1—S1	1.618 (3)
C7—H7A	0.9700	N1—H1N	0.841 (17)
C7—H7B	0.9700	N2—N3	1.380 (3)
C8—O3	1.224 (3)	N2—H2N	0.863 (18)
C8—N2	1.342 (4)	O1—S1	1.433 (2)
C9—N3	1.275 (4)	O2—S1	1.430 (2)
			
C2—C1—C6	120.8 (3)	C15—C10—C9	118.8 (3)
C2—C1—S1	120.2 (2)	C11—C10—C9	122.2 (3)
C6—C1—S1	119.0 (2)	C12—C11—C10	119.3 (3)
C1—C2—C3	119.9 (3)	C12—C11—H11	120.4
C1—C2—H2	120.0	C10—C11—H11	120.4
C3—C2—H2	120.0	C11—C12—C13	121.9 (4)
C4—C3—C2	119.1 (3)	C11—C12—Cl2	119.4 (3)
C4—C3—H3	120.4	C13—C12—Cl2	118.7 (3)
C2—C3—H3	120.4	C14—C13—C12	118.7 (3)
C3—C4—C5	121.4 (3)	C14—C13—H13	120.6
C3—C4—Cl1	119.8 (2)	C12—C13—H13	120.6
C5—C4—Cl1	118.8 (2)	C13—C14—C15	120.9 (4)
C4—C5—C6	119.5 (3)	C13—C14—H14	119.6
C4—C5—H5	120.3	C15—C14—H14	119.6
C6—C5—H5	120.3	C14—C15—C10	120.3 (4)
C5—C6—C1	119.3 (3)	C14—C15—H15	119.9
C5—C6—H6	120.4	C10—C15—H15	119.9
C1—C6—H6	120.4	C7—N1—S1	119.9 (2)
N1—C7—C8	111.0 (2)	C7—N1—H1N	116 (2)
N1—C7—H7A	109.4	S1—N1—H1N	112 (2)
C8—C7—H7A	109.4	C8—N2—N3	120.8 (2)
N1—C7—H7B	109.4	C8—N2—H2N	117 (2)
C8—C7—H7B	109.4	N3—N2—H2N	121 (2)
H7A—C7—H7B	108.0	C9—N3—N2	115.1 (3)
O3—C8—N2	121.4 (3)	O2—S1—O1	119.53 (13)
O3—C8—C7	121.1 (3)	O2—S1—N1	107.80 (13)
N2—C8—C7	117.5 (3)	O1—S1—N1	105.65 (13)
N3—C9—C10	121.6 (3)	O2—S1—C1	107.23 (13)
N3—C9—H9	119.2	O1—S1—C1	107.84 (13)
C10—C9—H9	119.2	N1—S1—C1	108.40 (13)
C15—C10—C11	119.0 (3)		
			
C6—C1—C2—C3	0.2 (5)	Cl2—C12—C13—C14	−178.0 (3)
S1—C1—C2—C3	−178.6 (3)	C12—C13—C14—C15	−0.4 (6)
C1—C2—C3—C4	−1.7 (5)	C13—C14—C15—C10	−0.2 (6)
C2—C3—C4—C5	1.5 (5)	C11—C10—C15—C14	0.3 (5)
C2—C3—C4—Cl1	−178.3 (3)	C9—C10—C15—C14	178.7 (3)
C3—C4—C5—C6	0.0 (5)	C8—C7—N1—S1	−107.2 (3)
Cl1—C4—C5—C6	179.8 (2)	O3—C8—N2—N3	176.8 (3)
C4—C5—C6—C1	−1.4 (5)	C7—C8—N2—N3	−3.8 (5)
C2—C1—C6—C5	1.3 (5)	C10—C9—N3—N2	179.1 (3)
S1—C1—C6—C5	−179.8 (2)	C8—N2—N3—C9	−169.5 (3)
N1—C7—C8—O3	−4.1 (4)	C7—N1—S1—O2	−50.2 (2)
N1—C7—C8—N2	176.5 (3)	C7—N1—S1—O1	−179.0 (2)
N3—C9—C10—C15	−170.4 (3)	C7—N1—S1—C1	65.6 (2)
N3—C9—C10—C11	8.0 (5)	C2—C1—S1—O2	−13.7 (3)
C15—C10—C11—C12	0.2 (5)	C6—C1—S1—O2	167.4 (2)
C9—C10—C11—C12	−178.2 (3)	C2—C1—S1—O1	116.3 (3)
C10—C11—C12—C13	−0.8 (6)	C6—C1—S1—O1	−62.6 (3)
C10—C11—C12—Cl2	178.1 (3)	C2—C1—S1—N1	−129.8 (3)
C11—C12—C13—C14	0.9 (6)	C6—C1—S1—N1	51.3 (3)

### (E)-4-Chloro-N-{2-[2-(3-chlorobenzylidene)hydrazinyl]-2-oxoethyl}benzenesulfonamide (II) Hydrogen-bond geometry (Å, º)

*Cg* is the centroid of the C1–C6 ring.

**Table:**

*D*—H···*A*	*D*—H	H···*A*	*D*···*A*	*D*—H···*A*
N1—H1*N*···O1^i^	0.84 (2)	2.23 (2)	3.041 (3)	161 (3)
N2—H2*N*···O3^ii^	0.86 (2)	1.97 (2)	2.828 (3)	171 (3)
C6—H6···O1^i^	0.93	2.53	3.422 (4)	161
C7—H7*B*···O2^iii^	0.97	2.47	3.434 (4)	172
C15—H15···O2^iv^	0.93	2.58	3.474 (4)	162
C14—H14···*Cg*^v^	0.93	2.84	3.675 (5)	150

Symmetry codes: (i) −*x*+1, −*y*, −*z*+2; (ii) −*x*+1, −*y*, −*z*+1; (iii) −*x*, −*y*, −*z*+2; (iv) *x*, *y*, *z*−1; (v) −*x*, −*y*, −*z*+1.

### (E)-4-Chloro-N-{2-[2-(4-chlorobenzylidene)hydrazinyl]-2-oxoethyl}benzenesulfonamide (III) Crystal data

**Table:**

C_15_H_13_Cl_2_N_3_O_3_S	*Z* = 2
*M**_r_* = 386.24	*F*(000) = 396
Triclinic, *P*1	*D*_x_ = 1.459 Mg m^−^^3^
*a* = 6.7234 (9) Å	Mo *K*α radiation, λ = 0.71073 Å
*b* = 10.281 (1) Å	Cell parameters from 1746 reflections
*c* = 13.611 (2) Å	θ = 2.9–27.9°
α = 74.98 (1)°	µ = 0.51 mm^−^^1^
β = 87.11 (1)°	*T* = 293 K
γ = 75.34 (1)°	Rod, colourless
*V* = 879.0 (2) Å^3^	0.46 × 0.42 × 0.20 mm

### (E)-4-Chloro-N-{2-[2-(4-chlorobenzylidene)hydrazinyl]-2-oxoethyl}benzenesulfonamide (III) Data collection

**Table:**

Oxford Diffraction Xcalibur with Sapphire CCD diffractometer	2600 reflections with *I* > 2σ(*I*)
Radiation source: fine-focus sealed tube	*R*_int_ = 0.019
Rotation method data acquisition using ω scans.	θ_max_ = 26.4°, θ_min_ = 2.9°
Absorption correction: multi-scan (CrysAlis RED, Oxford Diffraction, 2009)	*h* = −8→8
*T*_min_ = 0.801, *T*_max_ = 0.906	*k* = −12→12
5737 measured reflections	*l* = −13→16
3598 independent reflections	

### (E)-4-Chloro-N-{2-[2-(4-chlorobenzylidene)hydrazinyl]-2-oxoethyl}benzenesulfonamide (III) Refinement

**Table:**

Refinement on *F*^2^	Primary atom site location: structure-invariant direct methods
Least-squares matrix: full	Secondary atom site location: difference Fourier map
*R*[*F*^2^ > 2σ(*F*^2^)] = 0.048	Hydrogen site location: mixed
*wR*(*F*^2^) = 0.120	H atoms treated by a mixture of independent and constrained refinement
*S* = 1.03	*w* = 1/[σ^2^(*F*_o_^2^) + (0.0407*P*)^2^ + 0.5495*P*] where *P* = (*F*_o_^2^ + 2*F*_c_^2^)/3
3598 reflections	(Δ/σ)_max_ < 0.001
223 parameters	Δρ_max_ = 0.33 e Å^−^^3^
2 restraints	Δρ_min_ = −0.30 e Å^−^^3^

### (E)-4-Chloro-N-{2-[2-(4-chlorobenzylidene)hydrazinyl]-2-oxoethyl}benzenesulfonamide (III) Special details

**Table:**

Experimental. CrysAlis RED, Oxford Diffraction Ltd., 2009 Empirical absorption correction using spherical harmonics, implemented in SCALE3 ABSPACK scaling algorithm.
Geometry. All esds (except the esd in the dihedral angle between two l.s. planes) are estimated using the full covariance matrix. The cell esds are taken into account individually in the estimation of esds in distances, angles and torsion angles; correlations between esds in cell parameters are only used when they are defined by crystal symmetry. An approximate (isotropic) treatment of cell esds is used for estimating esds involving l.s. planes.
Refinement. Refinement of F^2^ against ALL reflections. The weighted R-factor wR and goodness of fit S are based on F^2^, conventional R-factors R are based on F, with F set to zero for negative F^2^. The threshold expression of F^2^ > 2sigma(F^2^) is used only for calculating R-factors(gt) etc. and is not relevant to the choice of reflections for refinement. R-factors based on F^2^ are statistically about twice as large as those based on F, and R- factors based on ALL data will be even larger.

### (E)-4-Chloro-N-{2-[2-(4-chlorobenzylidene)hydrazinyl]-2-oxoethyl}benzenesulfonamide (III) Fractional atomic coordinates and isotropic or equivalent isotropic displacement parameters (Å^2^)

**Table:**

	*x*	*y*	*z*	*U*_iso_*/*U*_eq_	
Cl1	−0.75370 (19)	0.81534 (16)	0.50474 (9)	0.1284 (5)	
Cl2	1.20248 (15)	0.42711 (14)	0.39838 (7)	0.1119 (4)	
S1	−0.27969 (10)	1.01993 (7)	0.11321 (5)	0.04891 (19)	
O1	−0.1031 (3)	1.05461 (19)	0.14290 (15)	0.0609 (5)	
O2	−0.4241 (3)	1.12132 (19)	0.04119 (15)	0.0628 (5)	
O3	−0.1441 (3)	0.66440 (19)	0.00307 (15)	0.0601 (5)	
N1	−0.1982 (3)	0.8899 (2)	0.06592 (17)	0.0518 (5)	
H1N	−0.285 (4)	0.868 (3)	0.038 (2)	0.062*	
N2	0.1718 (3)	0.5636 (2)	0.07085 (17)	0.0529 (5)	
H2N	0.179 (4)	0.490 (2)	0.050 (2)	0.063*	
N3	0.3257 (3)	0.5661 (2)	0.13326 (15)	0.0490 (5)	
C1	−0.4154 (4)	0.9674 (3)	0.2247 (2)	0.0487 (6)	
C2	−0.3286 (5)	0.9436 (4)	0.3189 (2)	0.0707 (8)	
H2	−0.199533	0.958848	0.324412	0.085*	
C3	−0.4330 (5)	0.8971 (4)	0.4049 (2)	0.0840 (10)	
H3	−0.374571	0.880441	0.468907	0.101*	
C4	−0.6231 (5)	0.8754 (4)	0.3961 (3)	0.0767 (9)	
C5	−0.7090 (5)	0.8990 (4)	0.3026 (3)	0.0909 (11)	
H5	−0.837854	0.883342	0.297257	0.109*	
C6	−0.6066 (5)	0.9456 (4)	0.2165 (2)	0.0773 (9)	
H6	−0.666116	0.962570	0.152735	0.093*	
C7	−0.0149 (4)	0.7821 (3)	0.10526 (19)	0.0471 (6)	
H7A	−0.022691	0.748572	0.178490	0.057*	
H7B	0.105960	0.818535	0.089975	0.057*	
C8	−0.0016 (4)	0.6659 (3)	0.05555 (18)	0.0466 (6)	
C9	0.4825 (4)	0.4633 (3)	0.1450 (2)	0.0521 (6)	
H9	0.486624	0.393537	0.112435	0.063*	
C10	0.6568 (4)	0.4535 (3)	0.20951 (19)	0.0480 (6)	
C11	0.6490 (4)	0.5479 (3)	0.2675 (2)	0.0606 (7)	
H11	0.530130	0.618121	0.266545	0.073*	
C12	0.8152 (5)	0.5385 (3)	0.3263 (2)	0.0682 (8)	
H12	0.808568	0.601525	0.365387	0.082*	
C13	0.9906 (4)	0.4354 (4)	0.3268 (2)	0.0648 (8)	
C14	1.0018 (4)	0.3406 (3)	0.2719 (2)	0.0692 (8)	
H14	1.120998	0.270324	0.273761	0.083*	
C15	0.8334 (4)	0.3497 (3)	0.2128 (2)	0.0596 (7)	
H15	0.840415	0.284985	0.175185	0.071*	

### (E)-4-Chloro-N-{2-[2-(4-chlorobenzylidene)hydrazinyl]-2-oxoethyl}benzenesulfonamide (III) Atomic displacement parameters (Å^2^)

**Table:**

	*U*^11^	*U*^22^	*U*^33^	*U*^12^	*U*^13^	*U*^23^
Cl1	0.1225 (9)	0.1721 (12)	0.0862 (7)	−0.0563 (9)	0.0375 (7)	−0.0130 (8)
Cl2	0.0737 (6)	0.1848 (12)	0.0779 (6)	−0.0439 (7)	−0.0247 (5)	−0.0185 (7)
S1	0.0460 (3)	0.0478 (4)	0.0565 (4)	−0.0123 (3)	−0.0034 (3)	−0.0181 (3)
O1	0.0516 (10)	0.0629 (12)	0.0799 (13)	−0.0225 (9)	−0.0016 (9)	−0.0303 (10)
O2	0.0616 (12)	0.0518 (11)	0.0695 (13)	−0.0097 (9)	−0.0111 (9)	−0.0076 (9)
O3	0.0520 (10)	0.0608 (11)	0.0742 (13)	−0.0063 (9)	−0.0165 (9)	−0.0329 (10)
N1	0.0454 (12)	0.0572 (13)	0.0591 (14)	−0.0092 (10)	−0.0070 (10)	−0.0277 (11)
N2	0.0500 (12)	0.0529 (13)	0.0604 (14)	−0.0070 (10)	−0.0122 (10)	−0.0258 (11)
N3	0.0473 (12)	0.0524 (13)	0.0495 (12)	−0.0115 (10)	−0.0077 (9)	−0.0161 (10)
C1	0.0438 (13)	0.0487 (14)	0.0566 (15)	−0.0078 (11)	−0.0012 (11)	−0.0215 (12)
C2	0.0552 (17)	0.103 (3)	0.0606 (18)	−0.0226 (16)	−0.0005 (14)	−0.0302 (18)
C3	0.076 (2)	0.119 (3)	0.0541 (19)	−0.019 (2)	−0.0019 (16)	−0.0212 (19)
C4	0.072 (2)	0.090 (2)	0.066 (2)	−0.0230 (18)	0.0196 (16)	−0.0157 (18)
C5	0.061 (2)	0.139 (3)	0.080 (2)	−0.044 (2)	0.0082 (17)	−0.023 (2)
C6	0.0577 (18)	0.118 (3)	0.0617 (19)	−0.0349 (18)	−0.0015 (15)	−0.0199 (19)
C7	0.0455 (13)	0.0516 (14)	0.0475 (14)	−0.0129 (11)	−0.0030 (11)	−0.0169 (12)
C8	0.0462 (13)	0.0511 (14)	0.0455 (14)	−0.0142 (11)	−0.0029 (11)	−0.0145 (11)
C9	0.0533 (15)	0.0527 (15)	0.0548 (15)	−0.0139 (12)	−0.0041 (12)	−0.0200 (12)
C10	0.0469 (13)	0.0483 (14)	0.0483 (14)	−0.0115 (11)	−0.0032 (11)	−0.0110 (11)
C11	0.0581 (16)	0.0583 (17)	0.0647 (18)	−0.0068 (13)	−0.0100 (13)	−0.0200 (14)
C12	0.075 (2)	0.077 (2)	0.0606 (18)	−0.0238 (17)	−0.0107 (15)	−0.0244 (16)
C13	0.0557 (17)	0.089 (2)	0.0481 (16)	−0.0261 (16)	−0.0076 (13)	−0.0056 (15)
C14	0.0499 (16)	0.075 (2)	0.0688 (19)	−0.0025 (14)	−0.0039 (14)	−0.0055 (16)
C15	0.0584 (16)	0.0557 (16)	0.0644 (18)	−0.0101 (13)	−0.0019 (13)	−0.0183 (14)

### (E)-4-Chloro-N-{2-[2-(4-chlorobenzylidene)hydrazinyl]-2-oxoethyl}benzenesulfonamide (III) Geometric parameters (Å, º)

**Table:**

Cl1—C4	1.735 (3)	C4—C5	1.362 (5)
Cl2—C13	1.738 (3)	C5—C6	1.368 (4)
S1—O1	1.4275 (18)	C5—H5	0.9300
S1—O2	1.4297 (19)	C6—H6	0.9300
S1—N1	1.594 (2)	C7—C8	1.500 (3)
S1—C1	1.764 (3)	C7—H7A	0.9700
O3—C8	1.229 (3)	C7—H7B	0.9700
N1—C7	1.450 (3)	C9—C10	1.467 (3)
N1—H1N	0.818 (17)	C9—H9	0.9300
N2—C8	1.341 (3)	C10—C15	1.375 (4)
N2—N3	1.380 (3)	C10—C11	1.390 (4)
N2—H2N	0.861 (17)	C11—C12	1.377 (4)
N3—C9	1.273 (3)	C11—H11	0.9300
C1—C2	1.373 (4)	C12—C13	1.371 (4)
C1—C6	1.374 (4)	C12—H12	0.9300
C2—C3	1.375 (4)	C13—C14	1.360 (4)
C2—H2	0.9300	C14—C15	1.392 (4)
C3—C4	1.369 (5)	C14—H14	0.9300
C3—H3	0.9300	C15—H15	0.9300
			
O1—S1—O2	119.70 (12)	N1—C7—C8	107.76 (19)
O1—S1—N1	107.02 (11)	N1—C7—H7A	110.2
O2—S1—N1	106.60 (12)	C8—C7—H7A	110.2
O1—S1—C1	107.38 (12)	N1—C7—H7B	110.2
O2—S1—C1	107.41 (12)	C8—C7—H7B	110.2
N1—S1—C1	108.32 (12)	H7A—C7—H7B	108.5
C7—N1—S1	121.73 (16)	O3—C8—N2	121.3 (2)
C7—N1—H1N	117 (2)	O3—C8—C7	121.3 (2)
S1—N1—H1N	116 (2)	N2—C8—C7	117.4 (2)
C8—N2—N3	119.7 (2)	N3—C9—C10	120.7 (2)
C8—N2—H2N	119.9 (19)	N3—C9—H9	119.7
N3—N2—H2N	119.6 (19)	C10—C9—H9	119.7
C9—N3—N2	115.3 (2)	C15—C10—C11	118.6 (2)
C2—C1—C6	120.1 (3)	C15—C10—C9	119.8 (2)
C2—C1—S1	120.5 (2)	C11—C10—C9	121.6 (2)
C6—C1—S1	119.3 (2)	C12—C11—C10	120.8 (3)
C1—C2—C3	119.8 (3)	C12—C11—H11	119.6
C1—C2—H2	120.1	C10—C11—H11	119.6
C3—C2—H2	120.1	C13—C12—C11	119.3 (3)
C4—C3—C2	119.7 (3)	C13—C12—H12	120.3
C4—C3—H3	120.1	C11—C12—H12	120.3
C2—C3—H3	120.1	C14—C13—C12	121.2 (3)
C5—C4—C3	120.4 (3)	C14—C13—Cl2	119.7 (3)
C5—C4—Cl1	119.9 (3)	C12—C13—Cl2	119.1 (2)
C3—C4—Cl1	119.7 (3)	C13—C14—C15	119.5 (3)
C4—C5—C6	120.3 (3)	C13—C14—H14	120.3
C4—C5—H5	119.8	C15—C14—H14	120.3
C6—C5—H5	119.8	C10—C15—C14	120.6 (3)
C5—C6—C1	119.7 (3)	C10—C15—H15	119.7
C5—C6—H6	120.2	C14—C15—H15	119.7
C1—C6—H6	120.2		
			
O1—S1—N1—C7	35.4 (2)	S1—C1—C6—C5	177.1 (3)
O2—S1—N1—C7	164.6 (2)	S1—N1—C7—C8	171.09 (18)
C1—S1—N1—C7	−80.1 (2)	N3—N2—C8—O3	−177.6 (2)
C8—N2—N3—C9	178.5 (2)	N3—N2—C8—C7	2.2 (4)
O1—S1—C1—C2	−10.8 (3)	N1—C7—C8—O3	−9.0 (3)
O2—S1—C1—C2	−140.8 (2)	N1—C7—C8—N2	171.1 (2)
N1—S1—C1—C2	104.5 (2)	N2—N3—C9—C10	180.0 (2)
O1—S1—C1—C6	171.5 (2)	N3—C9—C10—C15	−173.1 (3)
O2—S1—C1—C6	41.5 (3)	N3—C9—C10—C11	6.4 (4)
N1—S1—C1—C6	−73.3 (3)	C15—C10—C11—C12	0.6 (4)
C6—C1—C2—C3	0.4 (5)	C9—C10—C11—C12	−179.0 (3)
S1—C1—C2—C3	−177.3 (3)	C10—C11—C12—C13	0.5 (5)
C1—C2—C3—C4	−0.2 (5)	C11—C12—C13—C14	−1.4 (5)
C2—C3—C4—C5	0.3 (6)	C11—C12—C13—Cl2	178.2 (2)
C2—C3—C4—Cl1	179.5 (3)	C12—C13—C14—C15	1.1 (5)
C3—C4—C5—C6	−0.5 (6)	Cl2—C13—C14—C15	−178.5 (2)
Cl1—C4—C5—C6	−179.7 (3)	C11—C10—C15—C14	−0.9 (4)
C4—C5—C6—C1	0.6 (6)	C9—C10—C15—C14	178.7 (3)
C2—C1—C6—C5	−0.6 (5)	C13—C14—C15—C10	0.1 (5)

### (E)-4-Chloro-N-{2-[2-(4-chlorobenzylidene)hydrazinyl]-2-oxoethyl}benzenesulfonamide (III) Hydrogen-bond geometry (Å, º)

**Table:**

*D*—H···*A*	*D*—H	H···*A*	*D*···*A*	*D*—H···*A*
N1—H1*N*···O2^i^	0.82 (2)	2.25 (2)	3.032 (3)	162 (3)
N1—H1*N*···O3	0.82 (2)	2.23 (3)	2.609 (3)	109 (2)
N2—H2*N*···O3^ii^	0.86 (2)	1.98 (2)	2.829 (3)	170 (3)
C15—H15···O1^iii^	0.93	2.45	3.330 (3)	157

Symmetry codes: (i) −*x*−1, −*y*+2, −*z*; (ii) −*x*, −*y*+1, −*z*; (iii) *x*+1, *y*−1, *z*.
